# Simultaneous Quantification and Differentiation of *Streptococcus suis* Serotypes 2 and 9 by Quantitative Real-Time PCR, Evaluated in Tonsillar and Nasal Samples of Pigs

**DOI:** 10.3390/pathogens5030046

**Published:** 2016-06-30

**Authors:** Niels Dekker, Ineke Daemen, Koen Verstappen, Astrid de Greeff, Hilde Smith, Birgitta Duim

**Affiliations:** 1 Faculty of Veterinary Medicine, Department of Farm Animal Health, Utrecht University, P.O. Box 80.151, 3508 TD Utrecht, The Netherlands; a.j.j.m.daemen@uu.nl; 2 Faculty of Veterinary Medicine, Department of Infectious Diseases and Immunology, Utrecht University, P.O. Box 80.165, 3508 TD Utrecht, The Netherlands; k.verstappen@uu.nl (K.V.); b.duim@uu.nl (B.D.); 3 Central Veterinary Institute of Wageningen UR, P.O. Box 65, 8200 AB Lelystad, The Netherlands; astrid.degreeff@wur.nl (A.d.G.); hilde.smith@wur.nl (H.S.)

**Keywords:** *Streptococcus suis*, *S. suis*, serotype 2, serotype 9, *cps*, qPCR, pig, tonsil, nose

## Abstract

Invasive *Streptococcus suis* (*S. suis*) infections in pigs are often associated with serotypes 2 and 9. Mucosal sites of healthy pigs can be colonized with these serotypes, often multiple serotypes per pig. To unravel the contribution of these serotypes in pathogenesis and epidemiology, simultaneous quantification of serotypes is needed. A quantitative real-time PCR (qPCR) targeting c*ps2J* (serotypes 2 and 1/2) and *cps9H* (serotype 9) was evaluated with nasal and tonsillar samples from *S. suis* exposed pigs. qPCR specifically detected serotypes in all pig samples. The serotypes loads in pig samples estimated by qPCR showed, except for serotype 9 in tonsillar samples (correlation coefficient = 0.25), moderate to strong correlation with loads detected by culture (correlation coefficient > 0.65), and also in pigs exposed to both serotypes (correlation coefficient > 0.75). This qPCR is suitable for simultaneous differentiation and quantification of important *S. suis* serotypes.

## 1. Introduction

*Streptococcus suis* (*S. suis*) is a major pathogen in pigs. *S. suis* causes a wide variety of severe infections both in human and pigs including septicaemia, meningitis, and death [1,2]. In pig husbandry several control measures are implemented to restrict the animal welfare problems and economic losses caused by *S. suis*. As effective vaccines are lacking [1], high amounts of antimicrobials are often administered to pigs, which is undesirable because of antimicrobial resistance issues [3,4]. An accurate and rapid diagnosis of infection is essential for obtaining a reduction of antibiotic usage, and will contribute to our understanding of the pathogenesis and epidemiology of *S. suis* in pigs. In addition, a rapid diagnosis followed by suitable control measures may reduce (occupational) human exposure to *S. suis*. 

Based on differences in capsular polysaccharide (CPS) antigens detected with agglutination tests, 35 serotypes (1 to 34, and 1/2) have been classified for *S. suis* [5]. However, genetic analysis re-identified serotypes 32 and 34 as *Streptococcus orisratti*, and led to the suggestion that serotypes 20, 22, 26, and 33 do not belong to the *S. suis* taxon. Within the group of serological untypeable strains, recently 9 novel *cps* loci were described, as well as the proposal of a new serotype (serotype Chz) [6,7]. Cross-reactions have been described among some serotypes, e.g., serotypes 1 and 2 with serotype 1/2, and serotype 1 with serotype 14 [8,9]. This is due to the high similarity in *cps* loci of these serotype combinations, which cannot be distinguished by current PCR-based identification methods [5,10].

Among the confirmed serotypes isolated from diseased pigs worldwide, serotype 2 strains are highly prevalent [1,5]. The prevalence of specific serotypes, however, differs between regions, and can change in time. Besides serotype 2, in several European countries serotype 9 has become a highly prevalent or even the most prevalent serotype found among clinical isolates [5,11,12,13,14,15,16].

In the epidemiology of *S. suis* a key role is considered to be the carriage of *S. suis* on mucosal sites by healthy pigs [1]. In mucosal samples taken from different pigs on the same farm, and even within individual pigs several serotypes have been detected at the same time [17,18]. It is unknown what the epidemiological or pathogenic relevance is of simultaneous colonization of different serotypes. Further understanding is hampered by the lack of a diagnostic test that can detect and differentiate serotypes in animal samples, preferably quantitatively. A quantitative insight in the mucosal presence of individual serotypes will help to assess the contribution of these serotypes in causing mucosal infections [19]. In addition, the quantity of colonizing bacteria could be associated with infectiousness of animals, which is an important parameter in studying pathogen transmission [20].

Detection and quantification of *S. suis* in animal samples can be achieved by a (selective) bacterial culture procedure [21], but this has its limitations. The procedure of direct plating is labour-intensive and often hampered by overgrowth of other bacteria. Moreover, detection and quantification of more than one serotype is even more laborious and time consuming, and often with low sensitivity.

Other, more sensitive tests that have been used for detection and quantification of *S. suis* are immunomagnetic separation (IMS) [22] and polymerase chain reaction (PCR) [18,23]. A disadvantage of IMS is that it is expensive and laborious. Quantitative real-time PCRs (qPCR) for *S. suis* detection and quantification in pig, human or environmental samples have been published that target the 16S rRNA gene [19,24], the glutamate dehydrogenase (GDH) gene [25], or the fibronectin binding protein (FBPS) gene [26], or the serotypes 2 (and 1/2) specific *cps2J* gene [24,27]. However, no validated qPCRs have been reported for simultaneous detection of multiple serotypes in clinical samples.

Here we describe a multiplex qPCR for simultaneous detection, differentiation and quantification of *S. suis* serotype 2 (or 1/2) by targeting *cps2J*, and serotype 9 by targeting *cps9H*. Validation of the qPCR was performed with pure cultures and with nasal swab and tonsillar brushing samples of pigs exposed to either serotype 2 or 9 or both. The qPCR showed specific and sensitive detection of *S. suis* serotype 2 (and 1/2) and 9, and moderate to strong correlation with serotype loads detected by culture, except for detection of serotype 9 in tonsillar brushing samples of pigs exposed to serotype 9 that showed a weak to moderate correlation. 

## 2. Results

### 2.1. qPCR Validation with Pure Cultures

**Figure 1:**
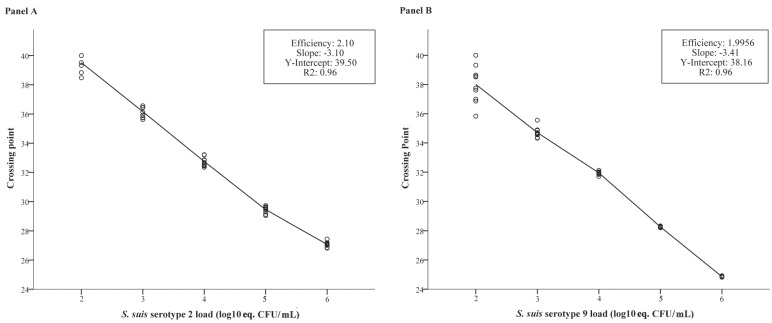
Standard curves of *cps2J*-qPCR (**panel A**) and *cps9H*-qPCR (**panel B**) obtained by serial dilutions of bacterial suspensions. The y-intercept indicates the expected crossing point (Cp) for a sample with a quantity equal to 1 eq. CFU/PCR reaction, i.e., 1 × 10^2^ eq. CFU/mL. The slope indicates the number of cycles between samples that differ 1.0 log10 eq. CFU /mL; a value of 3.3 is optimal. The R^2^ value indicates the close fit between the regression line of the standard curve and the individual Cp data points; a value of 1.00 indicates a perfect fit. The efficiency indicates the increase in copies per cycle; a value of 2 is optimal.

The qPCR was positive for cultures of bacterial strains containing the *cps2J* gene (i.e., *S. suis* serotypes 2 and 1/2) or the *cps9H* gene (i.e., serotype 9). The crossing point (Cp) values of these positive cultures ranged from Cp 23 to Cp 25 (i.e., 1 × 10^7^ to 1 × 10^6^ equivalent colony forming units per mL, eq. CFU/mL). Samples containing DNA of other *S. suis* serotypes or other bacterial species tested negative (Table 1). The standard curves of both the *cps2J-*PCR and the *cps9H-*PCR (Figure 1) detected spiked buffer samples that contained bacterial loads that could be expected in pig samples [28], i.e., between 1 × 10^2^ and 1 × 10^6^ eq. CFU/mL. High loads and titrations of serotype 9 DNA did not influence the ability to detect serotype 2, and high loads and titrations of serotype 2 DNA did not influence the ability to detect serotype 9. The in vitro analytical sensitivity of selective bacterial examination (SBE), determined by plating serial dilutions of pure cultures in saline, was calculated at 20 CFU/mL (200 CFU/sample) for tonsillar samples, and 20 CFU/mL (26 CFU/sample) for nasal samples. The analytical sensitivity of the qPCR was 1 eq. CFU per PCR reaction, both for serotype 2 (at Cp 40) and serotype 9 (at Cp 38). This resulted in a minimal detection limit of 40 eq. CFU /mL (i.e., 400 eq. CFU/sample) and 80 eq. CFU/mL (i.e., 104 eq. CFU/sample) for tonsillar and nasal samples, respectively.

### 2.2. qPCR Validation with Pig Samples

All tonsillar samples from uninfected pigs (*n* = 37) tested negative in the qPCR. This resulted in an estimated test specificity of 100% (95% CI: 0.92–1.00). All SBE positive tonsillar and nasal samples of pigs exposed to serotype 2 and/or serotype 9 tested positive in the qPCR for the respective serotype(s). This resulted in an estimated sensitivity of 100% for detection of serotype 2 in tonsillar samples (95% CI: 0.93–1.00) as well as in nasal samples (95% CI: 0.92–1.00). Similar results were obtained for serotype 9: in nasal and tonsillar samples the estimated sensitivity was 100% (95% CI: 0.92–1.00) and 100% (95% CI: 0.93–1.00), respectively.

Four tonsillar samples and 3 nasal samples of exposed pigs were negative by SBE, but tested positive by qPCR. Of these samples, 2 tonsillar samples and 3 nasal samples demonstrated relative low bacterial loads (<2.5 × 10^3^ eq. CFU/mL) of serotype 2 or serotype 9 or both types (Table S1). In the other 2 SBE-negative/PCR-positive tonsillar samples the loads were higher with 2.98 × 10^4^ and 1.55 × 10^5^ eq. CFU/mL, respectively. The internal control was inhibited in two samples (both nasal); these were excluded from statistical analysis.

**Figure 2:**
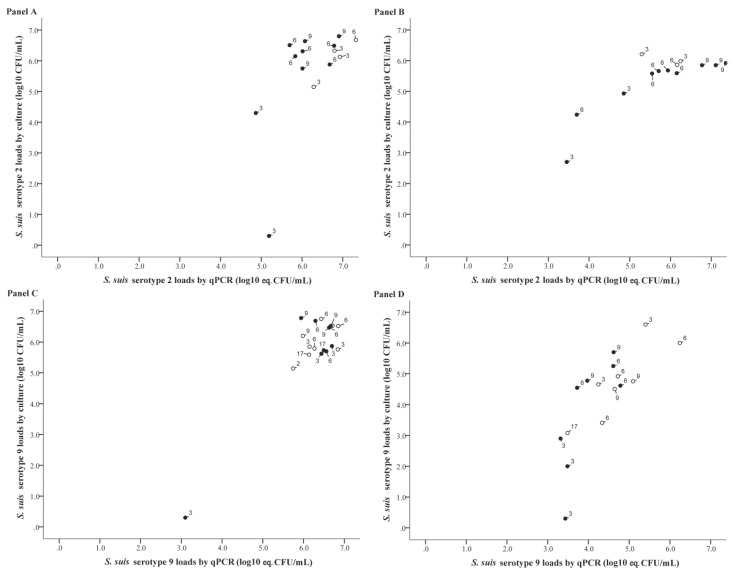
Correlation between the log10 of the number of colony forming units (CFU) determined by selective bacterial examination (SBE) and log 10 eq. CFU by qPCR in tonsillar samples (**panels A,C**) and in nasal samples (**panels B,D**) from pigs exposed to either *S. suis* serotype 2 (**panels A,B**) or serotype 9 (**panels C,D**). Pigs that were inoculated are marked by ○, and contact exposed pigs by ●. On the label of each individual point, its time point of sampling is presented (in days post inoculation).

The quantitative SBE and qPCR results of positive tonsillar and nasal samples taken from pigs exposed to one serotype (serotype 2 or 9) are visualized in Figure 2. Correlation analysis of positive samples from these pigs showed that in nasal samples the correlation coefficient between the loads determined by SBE and by qPCR was 0.82 (95% CI: 0.50–0.94) for serotype 2, and 0.78 (95% CI: 0.45–0.92) for serotype 9 (Table 2). For tonsillar samples these correlations were 0.68 (95% CI: 0.23–0.89) and 0.25 (95% CI: −0.26–0.65) for serotype 2 and serotype 9, respectively (Table 3). 

**Figure 3:**
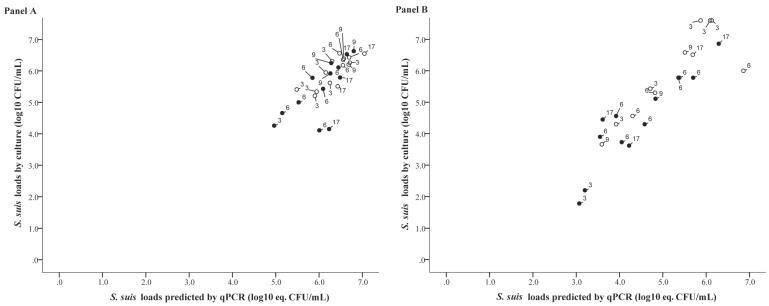
Correlation between the *S. suis* counts (log10 CFU/mL) determined by selective bacterial examination (SBE) and counts predicted by a linear mixed model with the qPCR results (log10 eq. CFU/mL) of tonsillar (**panel A**) and nasal samples (**panel B**) taken from pigs exposed to both serotypes 2 and 9 as input. The model was constructed with data of pigs colonized with either serotype 2 or 9. Pigs that were inoculated are marked by ○, and contact pigs by ●. On the label of each individual point, its time point of sampling is presented (in days post inoculation).

In Figure 3 the quantitative SBE results are shown against the *S. suis* loads predicted by the linear effects mixed model with the qPCR results of positive tonsillar (panel A) or nasal samples (panel B) taken from pigs exposed to both serotypes 2 or 9 as input. Statistical analysis showed that in samples of pigs colonized with both serotypes, the correlation between the SBE loads and the predicted loads based on the qPCR-results were 0.76 (95% CI: 0.54–0.88) and 0.90 (95% CI: 0.78–0.96) for tonsillar and nasal samples, respectively. Except for detection of serotype 9 in tonsillar and serotype 2 in nasal samples of pigs exposed to one serotype only, correlations did not show strong variation between inoculated and contact exposed pigs (Table 2 and Table 3; Figure 2 and Figure 3).

## 3. Discussion

In this study a qPCR for simultaneous detection and differentiation of *S. suis* serotypes 2 (and 1/2) and 9 in pig samples was developed. The qPCR performed well, both with pure cultures and samples of pigs that were exposed or non-exposed to *S. suis*. Test specificity and sensitivity relative to SBE were both 100%. Except for serotype 9 in tonsil and serotype 2 in nose of pigs exposed to only that serotype, there was a moderate to strong correlation between qPCR results and bacterial loads found by SBE in nasal and tonsillar samples of pigs colonized with one or both serotypes. This qPCR is a useful tool for studies that need (relative) quantification of serotype(s) loads in animal samples, like pathogenesis and epidemiological studies.

Several PCR tests have been described for *S. suis*. Relative to single target designed PCR tests, new multiplex tests were increasingly described in the last years [23,38,39,40,41]. Among these multiplex tests, however, most by far are non-real-time and cannot quantify bacterial loads accurately. Moreover, several tests were validated for identification of cultured *S. suis* strains, and not for direct detection in pig or human samples [38,39,40,41]. Some real-time PCR tests have been developed that focused on detection of *S. suis* in pig blood, or serotype 2 specific in human cerebrospinal fluid [25,27], and one targeting *S. suis* 16S rRNA gene in jejunal mucosal and digesta samples of pigs [19]. In a recent study qPCR targeting both 16S rRNA and serotype 2 specific *cps2J* genes was carried out on air samples of swine confinement buildings and human nasal swab samples. In that study, however, the qPCR tests were performed separately for the different targets, and not in multiplex design [24]. For serotype 9 one real-time PCR was published, but it was only applied to identify pure cultures, and not to quantify loads [28]. The qPCR test presented here can both detect and quantify the serotypes 2 and 9, and was validated for detection in samples from pigs with a commensal bacterial flora. 

The specificity of the qPCR for both serotype 2 (or 1/2) and serotype 9 was high. This is in accordance to other PCRs targeting *S. suis cps* genes [38,39,41]. Similar to other PCRs no differentiation could be made between serotypes 2 and 1/2, because no unique *cps* genes have been identified for serotype 1/2 so far [5,10]. Consequently, in samples that can contain multiple serotypes (e.g., mucosal samples) [17], it is not possible to differentiate between presence of serotype 1/2 solely, and serotypes 1/2 and/or 2 together. Nevertheless, the current qPCR could be a useful tool in epidemiological studies on serotype 9 in field pigs, and for combined detection of serotypes 2 and 9 in experiments with pigs with a known *S. suis* status. Furthermore, this qPCR can be applied for surveillance on absence of serotypes in a pig population, and after evaluation in field settings, may be indicative for defining a *S. suis* “health status” of farms.

The minimal serotype load that could be detected by qPCR was slightly higher than that of the SBE procedure. Nevertheless, some pig samples that were negative in SBE tested positive in the PCR, and no SBE positive samples were PCR negative. A likely explanation is that qPCR detected DNA of dead or otherwise non-vital bacteria that could lead to a slight overestimation of CFU in samples. Loss of bacterial vitality due to inadequate transport and storing conditions may result in an underestimation of CFU in culture. Overall, qPCR seems more sensitive than SBE in detecting *S. suis* in tonsillar and nasal samples of pigs. This conclusion is based on results of pigs that were raised under high biosecurity conditions, and with a controlled exposure to only one or two different *S. suis* serotypes. Under field conditions pigs might, however, be colonized with more serotypes and/or other bacterial species [17,18]. The diverse bacterial flora will limit the detection of a single serotype with culture, but will not affect detection with qPCR. As a consequence the qPCR sensitivity will likely be higher than the sensitivity of SBE under field conditions.

Compared to several other *S. suis* serotype-specific PCR tests, the presented qPCR was able to detect lower loads (detection limit: 80 and 40 eq. CFU/mL in nose and tonsil, respectively). In PCR studies on detection in clinical human or pig samples by targeting *cps2J* the minimal detection limit ranged from 28 eq. CFU/mL to about 300 eq. CFU/mL [18,23,27]. In one of those studies the limit (i.e., 280 eq. CFU/mL) was determined with in vitro inoculated tonsillar biopsy specimens [23]. We used serially diluted bacterial suspensions for determination of the detection limit and it can be hypothesized that in tonsillar biopsies or brushing samples the minimal detection limit of our qPCR would be higher, due to inhibitory factors in tissue materials/mucosa [42]. However, the internal PCR control did not indicate inhibition in any of the tonsillar samples.

qPCR inhibition was detected in two pig nasal swabs. That was further confirmed by the observation that the loads were average to high by culture, and extremely low by qPCR. Thus, the internal control facilitated the interpretation of the qPCR results, and may thereby promote reduction of the number of false negatives. This might be even more important when testing more complex samples, like mucosal samples of field pigs or faecal samples [19,43], in which the presence of high amounts of other microorganisms and/or other inhibitory substances may contribute to PCR reaction inhibition [42,44].

One of the main objectives for developing this qPCR was to be able to quantify the bacterial load of serotype 2 and/or 9 in a sample in an efficient way. In tonsils of pigs exposed to serotype 2 only or to both serotypes 2 and 9, the loads found by SBE strongly correlated to the loads determined by qPCR. Remarkably, in pigs exposed to serotype 9 only, and especially in contact-exposed ones, this correlation was weak (Table 3). Likely that was due to the narrow range of loads observed in this group (Figure 2). However, based on the in vitro validation experiments (Figure 1), on tonsillar samples of pigs exposed to both types that contained higher serotype 9 than serotype 2 loads (Table S1), and on the observed strong correlation between SBE and qPCR for serotype 9 in nasal swab samples (Table 2), the results indicate that it is likely that serotype 9 in tonsillar samples qPCR would also correlate well with SBE when samples with a broader range of loads are tested. In nasal samples a strong correlation was also observed between qPCR and SBE loads, except for pigs inoculated with serotype 2 only. In that group the results were hampered by a low number of samples (*n* = 3). In this context it has to be stated that correlation analysis was mainly based on loads above 10^4^ CFU/mL, and only in the in vitro validation loads of repeated samples with <10^4^ CFU/mL were tested; these low loads correlated well with qPCR results (Figure 1). Nevertheless, for nasal and tonsillar samples within a lower load range the correlation should be considered with care. On the other side of the load spectrum, unexpected high loads [28] were observed that exceeded the highest load of the standard curve (i.e., >10^6^ eq. CFU/mL). The precision of quantitative real-time PCR is particularly low when the initial target is low. PCR efficiency may be more variable at low copy numbers and therefore we studied this extensively in the in vitro validation (Figure 1). In contrast, quantification with higher amounts of target is generally more consistent [45], as shown in Figure 1. We therefore think it is reasonable to increase the logistic maximum of the standard curve progressively above 10^6^ eq. CFU/mL. In this extrapolation it is assumed that the ratio between viable and dead bacterial cells will be comparable at higher loads, which is likely for the standard curve as each series consisted of dilutions of the same original cell culture. Whether this assumption applies to pig samples has not been studied for *S. suis*. It can be hypothesized that the proportions of dead and live *S. suis* cells may vary at different loads, e.g., by variation in immune reactivity. In our study, however, the moderate to strong correlation between qPCR and SBE for several sample sets that include samples with very high loads (>10^6^ eq. CFU/mL), i.e., tonsillar samples after serotype 2 infection and both tonsillar and nasal samples after combined infection with serotypes 2 and 9, suggests that at least for these sample types and serotypes this variation in proportions between dead and live bacteria were not extensive. 

In conclusion, implementation of *cps2J* and *cps9H* detection in a multiplex qPCR design, as performed here, provides efficient estimation of the relative load of two serotypes present in samples, and the results indicate that this qPCR is suitable to estimate the quantity of serotype 2 loads in tonsillar as well as nasal samples, and of serotype 9 loads in nasal samples and less adequately in tonsillar samples.

Although efforts have been made to understand the pathogenesis and epidemiology of *S. suis* [1,46,47,48], many questions remain. For instance, studies with other streptococci (e.g., *Streptococcus pneumoniae*) have shown that when multiple serotypes are simultaneously present on host mucosa, the distinct serotypes affect each other in e.g., colonization efficiency [49,50,51]. Similar to these streptococci, it has been reported for *S. suis* that multiple serotypes can colonize an individual at the same time [17,18]. It is, however, unknown if and to what extent different *S. suis* serotypes influence each other in colonization, in disease induction, or in spread amongst individuals. The possibility of this qPCR to differentiate serotypes 2 and 9, and to quantify their individual loads could, together with its low detection limit, contribute to further understanding of these aspects. In addition, the qPCR test can also be useful for clinical diagnostics, evaluation of disease control measures and possibly surveillance of farm status involving these *S. suis* serotypes.

## 4. Experimental Section

### 4.1. Bacterial Strains 

*S. suis* serotype 9 strain 7997 [11] and *S. suis* serotype 2 strain 10 [35] were used to optimize the PCR protocol, and for inoculation of pigs. The specificity of the qPCR test was evaluated using cultures of reference strains and field strains of *S. suis*, and strains of 11 other bacterial species that are considered to colonize the oropharyngeal and nasal mucosae of pigs (Table 1). The *S. suis* strains covered the originally described serotypes 1–34 and 1/2; the serotype of each strain had been determined by slide agglutination with specific hyperimmune sera (CVI, Lelystad, The Netherlands).

Bacteria were grown overnight at 37 °C and 5% CO_2_ on blood agar containing 5% (volume/volume) sheep blood (BioTrading, Mijdrecht, The Netherlands) or heart infusion agar with 5% (v/v) sheep blood and, depending on their β-nicotinamide adenine dinucleotide (NAD) requirement, supplemented with 0.5% NAD (AppliChem GmbH, Darmstadt, Germany). One colony of each strain from an overnight culture was suspended in 0.5 mL 1 × TE (10 mM Tris-HCL, 1 mM EDTA) for DNA-isolation.

### 4.2. Primers and Probes

The qPCR for both serotypes was developed with specific sequences, *cps2J* for serotypes 2 (and 1/2), and *cps9H* for serotype 9 as described by Smith et al. [52]. For the serotype 9—qPCR the primer sequences were modified and for the serotype 2—qPCR two new primers were designed (Table 4). Probe sequences were developed by BLAST homology search with available sequences in GenBank on March 15th 2016. The *cps9H* probe contained a 5′-6FAM and 3′-BHQ1, and the *cps2J* probe contained an 5′-ATTO 532 (Yakima Yellow replacement) and 3′-BHQ1. Possible PCR inhibition by components in the clinical samples was monitored with an internal control consisting of two primers and a hydrolysis probe targeting pUC19 [53]. The probe contained a 5′-Cy5 and a 3′-BHQ2. All used oligonucleotides were obtained from Biolegio BV (Nijmegen, The Netherlands) and are shown in Table 4.

### 4.3. Multiplex qPCR

For each individual PCR reaction the optimal primer concentration, between 100 to 900 nM, was determined. With the optimal primer concentration the probe concentrations ranging from 50 to 250 nM were evaluated. The optimal concentrations were combined in the multiplex PCR and the efficiency for detection of each target was determined. As PCR template, spiked *S. suis* in TE buffer consisting of a 10-fold serial dilution with 10^4^ to 1 eq. CFU/PCR (i.e., 10^6^ to 10^2^ eq. CFU/mL) was used. DNA was isolated with InstaGene™ Matrix (Bio-Rad, Hercules, CA, USA) according to the manufacturer’s instructions, with some minor modifications as recently published [54].

Of the internal control, 10 pUC19 copies were consistently detected without interference of the *cps9H* or *cps2J* detection.

A primers/probes-mix consisting of 300 nM of each *cps2J-* and *cps9H-*primer, 125 nM of the *cps9H-*probe, 150 nM of the *cps2J-*probe, and 100 nM of each pUC19 primer and probe was used. The PCR mixture of 20 μL contained 10 μL LC480 Probes Master (2×) (Roche Diagnostics, Almere, The Netherlands), 1 µL of the primers/probes-mix, 4 µL PCR-grade water (Roche Diagnostics, Basel, Switzerland) and 5 µL template DNA. DNA-amplification was carried out in a LightCycler^®^ 480-II Instrument (Roche Diagnostics GmbH, Mannheim, Germany). In each run a negative extraction control from each DNA isolation run, an amplification control (water) and 2 positive controls consisting of DNA from serotype 9 strain 7997 and serotype 2 strain 10 were included. The PCR conditions were as follows: 95 °C for 10 min, 45 cycles of 95 °C for 10 s, 58 °C for 30 s, and 72 °C for 30 s, and a final extension at 72 °C for 8 min.

### 4.4. Standard Curves for Quantification

DNA extractions of 10-fold serial dilutions of pure cultures of each serotype, ranging from 1 to 10^4^ eq. CFU/PCR (i.e., 10^2^ to 10^6^ eq. CFU/mL), were used ten times for construction of the standard curves to calculate the *S. suis* serotype eq. CFU/PCR. With the multiplex qPCR the loads of each serotype in samples was determined using the LightCycler^®^ 480 algorithm. In each PCR a standard DNA concentration of each serotype was included to normalise the standard curve for run-to-run variation.

### 4.5. qPCR Validation with Pig Samples

To validate the qPCR test, tonsillar and nasal samples were collected from Landrace x Yorkshire pigs that were obtained by caesarean section from sows from the farrowing farm of the Faculty of Veterinary Medicine (Utrecht, The Netherlands), and were raised colostrum-deprived as described before [28]. These pigs were also part of another study, that did not interfere with the detection of *S. suis* in tonsillar and nasal samples by SBE or qPCR. The animal experiments were approved by the Animal Care and Ethics Committee of Utrecht University, in accordance with the Dutch law on animal testing (approval number DEC 2011.II.08.125).

The pigs were successively housed in isolators (0–4 weeks of age) and in a ground floor pen (weeks 5 and 6). At the end of week 6 they were randomly assigned to one of the three groups, that differed only in *S. suis* serotypes the pigs would be exposed to: serotype 2 (group 1), or serotype 9 (group 2), or both serotypes (group 3). The groups, each including 12 piglets, were housed in separate units. In these units the pigs were housed pairwise in boxes. The boxes had completely closed, multiplex walls (height: 80 cm), plasticised iron grid floor, rubber lying area, feeding trough and drinking nipple, and bite sticks as environmental enrichment. Strict biosecurity measures were implemented to avoid contamination of the pigs with other serotypes than they were experimentally exposed to, or with other pathogens. Each unit had its own air filtration system and airlocks, and animal caretakers had to wear unit-specific clothes and had to take a shower after visiting the pigs. The pigs were, however, not considered gnotobiotic as they were exposed to, e.g., (non-pathogenic) bacteria present in the feed, and in pig samples the presence of other bacteria than those that could grow on the selective culture media was not monitored.

At day 41 in each pair one pig was intranasally inoculated with *S. suis*. These pigs received 5 mL containing 1 × 10^9^ CFU serotype 2 (group 1) or 1 × 10^9^ CFU serotype 9 (group 2) or 1 × 10^9^ CFU serotype 2 and 1 × 10^9^ CFU serotype 9 (group 3), as described previously [28]. It was expected that the non-inoculated pigs, referred to as “contact pigs”, would be colonized within 24 to 48 hours post exposure to their inoculated pen-mates [28].

Before inoculation, tonsillar samples were taken (at 5 days before inoculation) (*n* = 37). After inoculation both tonsillar (*n* = 63) and nasal samples (*n* = 57) were taken, distributed over inoculated (29 tonsillar, and 23 nasal samples) and contact pigs (34 tonsillar, and 32 nasal samples). Those samplings were performed at days 3, 6, 9 and 17 post inoculation, to cover different stages of infection. For logistical and financial reasons we sampled 24 of the 36 piglets, and not longer than 17 days after inoculation. 

Tonsillar samples were obtained by brushing both palatine tonsillar areas for 3 s each with a sterile toothbrush. Nasal samples were obtained by twirling a swab (type MW102, MedicalWire and Equipment, Corsham, UK) in one nostril for 3 s. The brush and swab heads were put in separate sterile tubes containing 10 mL (tonsil) or 1.3 mL (nose) saline solution and transported to the laboratory. One mL of the tonsillar brush and 0.5 mL of the nasal swab suspension were stored at −20 °C and used for DNA-isolation and subsequent qPCR. One hundred µL of each suspension was used to prepare a 10-fold serial dilution (from undiluted to 1:10^3^) for SBE. All dilutions were plated on selective agar plates containing Columbia agar, 6% sheep blood, 0.2 μg/mL crystal violet and colistin/oxolinic acid (BioTrading, Mijdrecht, The Netherlands), and incubated at 37 °C and 5% CO_2_. Colonies suspected of *S. suis* on the basis of colony morphology were counted, and per plate two isolates were subcultured and tested for amylase activity. Isolates that showed amylase activity were serotyped by slide agglutination with *S. suis* serotype specific hyperimmune sera against serotypes 1, 2 and 9 (CVI, The Netherlands).

### 4.6. Statistical Analysis

The data were statistically analysed using R version 2.13.0 (R Foundation for Statistical Computing, Vienna, Austria) and IBM SPSS Statistics for Windows 20.0 (IBM Corp., Armonk, NY, USA). Bacterial loads were expressed in CFU per mL of the original sample suspension (CFU/mL) for SBE and in equivalent CFU per mL of the original sample suspension (eq. CFU/mL) for qPCR.

If, in streaks of the highest sample dilution (1:10^3^), the number of colonies per agar plate exceeded 200, it was qualified as uncountable. For these samples (i.e., 5 tonsil brushing and 5 nasal swab samples) 4 × 10^7^ CFU/mL was used in the analysis; it did not influence the conclusions if the maximum detection limit of SBE, i.e., 4 × 10^6^ CFU/mL, was used.

The qPCR loads higher than the highest concentration in the standard curve (i.e., 10^4^ eq. CFU/PCR, which is similar to 1 × 10^6^ eq. CFU/mL) were extrapolated using the algorithm of the LightCycler^®^ 480 Instrument. Samples were retested if the Cp value of the internal control exceeded the determined cut-off value (Cp 29) of either tonsillar or nasal samples, and if retesting led to the same result they were excluded from quantitative loads analysis, and when the *cps9H* and/or *cps2J* reaction were negative also from sensitivity/specificity analysis.

Loads were log10 transformed to normalize the data. The relation between the quantitative results of SBE and qPCR in pig samples was determined by performing a conditional analysis on the SBE positive samples. In pigs colonized with either serotype 2 or serotype 9 (group 1 and 2), correlation between SBE and qPCR was determined. In samples of pigs colonized with both serotypes the load per serotype could be determined by qPCR, but not by SBE. For analysis of the relation between the qPCR and SBE results for these samples, linear mixed effects model analysis was used [55]. Based on qPCR and SBE results of samples of pigs colonized with either serotype 2 or serotype 9 (group 1 and 2), a linear mixed effects model with the assumption of a normal distribution for the outcome was constructed. The load of *S. suis* (in log10 CFU/sample) was used as dependent variable. The qPCR-result (log10 eq. CFU/mL), serotype (2 or 9) and their interaction were included as fixed effects. The assumptions of normality of residuals and equal variances of the final model were confirmed by visual inspection of quantile-quantile plots, plots of standardized residuals against predicted values, and plots of standardized residuals against all predictor variables. The final model was used to predict the bacterial loads per serotype based on the qPCR-results of the respective serotypes in the samples taken from pigs colonized with both serotypes (group 3). For evaluation of the quantitative relation between qPCR and SBE in these samples, the Pearson’s correlation between the total *S. suis* load (serotype 2 and 9) predicted by the model and the total *S. suis* load found by SBE was determined. Within all three groups correlation was determined for contact and inoculated pigs together as well as separately.

## Figures and Tables

**Table 1:** Bacterial strains for testing the specificity of the *cps2J* and *cps9H* qPCR.

Species	Serotype	Strain	Source/Reference	qPCR Result
*Streptococcus suis*	1/2	Reference, 2651	CVI [29]	+
	2	Reference, 735	CVI [29]	+
	9	Reference, 22083	CVI [8]	+
	1; 3–8; 10–34	Reference *	CVI [8,29,30,31,32]	-
	1	6388	CVI [33]	-
	1	C160	CVI [34]	-
	1	C187	CVI [34]	-
	1	6112	CVI [33]	-
	1	6555 (NCTC428)	CVI	-
	2	D282	CVI [35]	+
	2	22	CVI [35]	+
	2	17	CVI	+
	2	10	CVI [35]	+
	2	T15	CVI [36]	+
	2	89–1591	CVI [37]	+
	7	15009	CVI [34]	-
	7	C126	CVI [34]	-
	7	7917	CVI [34]	-
	7	8039	CVI [34]	-
	7	7711	CVI [34]	-
	9	7997	CVI [11]	+
	9	7709	CVI [34]	+
	9	C132	CVI [34]	+
	9	8017	CVI [34]	+
	9	5973	CVI [34]	+
	9	7998	CVI [34]	+
*Actinobacillus pleuropneumoniae*	1	S4074	CVI	-
	2	1536	CVI	-
	9	13261	CVI	-
*Actinobacillus lignieresii*		Field strain	FVM	-
*Actinobacillus suis*		Field strain	FVM	-
*Bordetella bronchiseptica*		Field strain	FVM	-
*Enterococcus faecium*		DSM 7134	Nutreco	-
*Escherichia coli*		Field strain	FVM	-
*Erysipelothrix rhusiopathiae*		Field strain	FVM	-
*Pasteurella multocida*		Field strain	FVM	-
*Pasteurella pneumotropica*		Field strain	CVI	-
*Staphylococcus aureus*		Field strain	FVM	-
*Staphylococcus hyicus*		Field strain	FVM	-

CVI, Central Veterinary Institute, The Netherlands; FVM, Faculty of Veterinary Medicine, Utrecht University, The Netherlands. * Reference strains used for these serotypes (st): 428 (st1), 4961 (st3), 6407 (st4), 11538 (st5), 2524 (st6), 8074 (st7), 14636 (st8), 4417 (st10), 12814 (st11), 8830 (st12), 10581 (st13), 13730 (st14), NCTC 10446 (st15), 2726 (st16), 93A (st17), NT77 (st18), 42A (st19), 86–5192 (st20), 14A (st21), 88-1861 (st22), 89-2479 (st23), 88-5299A (st24), 89-3576-3 (st25), 89-4109-1 (st26), 89-5259 (st27), 89-590 (st28), 92-1191 (st29), 92-1400 (st30), 92-4172 (st31), EA1172.91 (st32), EA1832.92 (st33), 92-2742 (st34).

**Table 2:** Correlation coefficients between *Streptococcus suis* loads determined by selective bacterial examination (SBE) and predicted based on qPCR results of nasal samples taken from pigs that differ in serotype exposure and mode of exposure.

Exposure to *S. suis*		Correlation Coefficient SBE (log 10 CFU/mL) vs. Predicted qPCR (log10 eq. CFU/mL)	95% CI	N Samples
Exposure Mode	Serotype			
Inoculated	2	−0.91	NP; *p* = 0.27	3
Contact	2	0.88	0.59–0.97	10
Inoculated + Contact	2	0.82	0.45–0.92	13
Inoculated	9	0.78	0.16–0.96	8
Contact	9	0.80	0.11–0.97	7
Inoculated + Contact	9	0.78	0.45–0.92	15
Inoculated	2 + 9	0.85	0.53–0.96	12
Contact	2 + 9	0.91	0.71–0.97	12
Inoculated + Contact	2 + 9	0.90	0.78–0.96	24

NP: not possible to calculate the 95% CI interval due to the limited number of samples; the *p*-value refers to the null hypothesis that the correlation coefficient between SBE en predicted qPCR is 0.

**Table 3:** Correlation coefficients between *Streptococcus suis* loads determined by selective bacterial examination (SBE) and predicted based on qPCR results of tonsillar samples taken from pigs that differ in serotype exposure and mode of exposure.

Exposure to *S. suis*		Correlation Coefficient SBE (log 10 CFU/mL) vs. Predicted qPCR (log10 eq. CFU/mL)	95% CI	N Samples
Exposure Mode	Serotype			
Inoculated	2	0.95	0.43–0.99	5
Contact	2	0.72	0.10–0.93	9
Inoculated + Contact	2	0.68	0.23–0.89	14
Inoculated	9	0.60	−0.11–0.90	9
Contact	9	−0.47	−0.88–0.34	8
Inoculated + Contact	9	0.25	−0.26–0.65	17
Inoculated	2 + 9	0.80	0.49–0.93	15
Contact	2 + 9	0.70	0.24–0.90	13
Inoculated + Contact	2 + 9	0.76	0.54–0.88	28

**Table 4:** Primers and probes used to construct the internally controlled qPCR.

Name	Sequence (5′-3′)	Product Size	Reference
*cps*2J-FW	ACGCAGAGCAAGATGGTAGAATAA	135 bp	This study
*cps*2J-REV	TGCCGTCAACAATATCATCAGAA		This study
*cps*2J-probe	CAAACGCAAGGAATTACGGTATC		This study ^a^
*cps*9H-FW	CAAAGTTAGTTCAGGAAGGAATAGTCT	140 bp	[28]
*cps*9H-REV	CCGAAGTATCTGGGCTACTG		[28]
*cps*9H-probe	TTCAGATCAAGATGATATTTGGGACT		[28]
pUC19FW	GAGACGGTCACAGCTTGTCT	184 bp	This study ^b^
pUC19REV	TGATGCGGTATTTTCTCCTT		This study
pUC probe	CGGCATCAGAGCAGATTGTA		This study

^a^ Sequence is used as forward primer by Smith et al. [52]; ^b^ Developed according to Abdulmawjood et al. [53].

## Supplementary Materials

The following are available online at www.mdpi.com/2076-0817/5/3/46/s1, Table S1: Quantitative bacteriological and qPCR data of individual nasal and tonsillar samples.
